# Supplementary material for: An open-source framework for large-scale, flexible evaluation of biomedical text mining systems
Source: J Biomed Discov Collab. 2008 Jan 29;3:1. doi: 10.1186/1747-5333-3-1 (PMC2276192; doi:10.1186/1747-5333-3-1)
Supplement: Additional file 1 — Evaluation platform source code. The additional file contains the software evaluation framework discussed in this paper. The most current release can be downloaded from SourceForge.net [1]. See the accompanying README file for instructions on installation and use. Also included in the distribution is a collection of UIMA wrappers for some commonly used BioNLP tools and annotated corpora. [file 1747-5333-3-1-S1.zip › bionlp_uima_repository-1.0.1/docs/api/src-html/edu/uchsc/ccp/util/nlp/mention/MentionSortComparator.html]

```
001    package edu.uchsc.ccp.util.nlp.mention;
002    
003    import java.util.Comparator;
004    
005    /**
006     * Allows lists of mentions to be sorted by Collections.sort() so that the getSingleLineRepresentation() methods output the same string no matter what
007     * order the mentions were added
008     * 
009     * @author williamb
010     * 
011     */
012    public class MentionSortComparator implements Comparator<Mention> {
013    
014        public int compare(Mention m1, Mention m2) {
015                return m1.getSingleLineRepresentation().compareTo(m2.getSingleLineRepresentation());
016        }
017    
018    }
```
